# Potential of Marine Bacterial Metalloprotease A69 in the Preparation of Peanut Peptides with Angiotensin-Converting Enzyme (ACE)-Inhibitory and Antioxidant Properties

**DOI:** 10.3390/md22070305

**Published:** 2024-06-29

**Authors:** Wen-Jie Cao, Rui Liu, Wen-Xiao Zhao, Jian Li, Yan Wang, Xiao-Jie Yuan, Hui-Lin Wang, Yu-Zhong Zhang, Xiu-Lan Chen, Yu-Qiang Zhang

**Affiliations:** 1 State Key Laboratory of Microbial Technology, Marine Biotechnology Research Center, Shandong University, Qingdao 266237, China; 202232574@mail.sdu.edu.cn (W.-J.C.); liurui7317@163.com (R.L.); 202132652@mail.sdu.edu.cn (W.-X.Z.); fighter1216217564@163.com (J.L.); 201611778@mail.sdu.edu.cn (Y.W.); yuanxj0906@163.com (X.-J.Y.); whlsd822@163.com (H.-L.W.); zhangyz@sdu.edu.cn (Y.-Z.Z.); 2 Frontiers Science Center for Deep Ocean Multispheres and Earth System, College of Marine Life Sciences, Ocean University of China, Qingdao 266003, China; 3 Joint Research Center for Marine Microbial Science and Technology of Shandong University and Ocean University of China, Qingdao 266237, China; 4 Laboratory for Marine Biology and Biotechnology, Qingdao Marine Science and Technology Center, Qingdao 266237, China

**Keywords:** marine bacterial metalloprotease A69, peanut peptides, angiotensin-converting enzyme inhibitory activity, antioxidant activity

## Abstract

Marine bacterial proteases have rarely been used to produce bioactive peptides, although many have been reported. This study aims to evaluate the potential of the marine bacterial metalloprotease A69 from recombinant *Bacillus subtilis* in the preparation of peanut peptides (PPs) with antioxidant activity and angiotensin-converting enzyme (ACE)-inhibitory activity. Based on the optimization of the hydrolysis parameters of protease A69, a process for PPs preparation was set up in which the peanut protein was hydrolyzed by A69 at 3000 U g^−1^ and 60 °C, pH 7.0 for 4 h. The prepared PPs exhibited a high content of peptides with molecular weights lower than 1000 Da (>80%) and 3000 Da (>95%) and contained 17 kinds of amino acids. Moreover, the PPs displayed elevated scavenging of hydroxyl radical and 1,1-diphenyl-2-picryl-hydrazyl radical, with IC_50_ values of 1.50 mg mL^−1^ and 1.66 mg mL^−1^, respectively, indicating the good antioxidant activity of the PPs. The PPs also showed remarkable ACE-inhibitory activity, with an IC_50_ value of 0.71 mg mL^−1^. By liquid chromatography mass spectrometry analysis, the sequences of 19 ACE inhibitory peptides and 15 antioxidant peptides were identified from the PPs. These results indicate that the prepared PPs have a good nutritional value, as well as good antioxidant and antihypertensive effects, and that the marine bacterial metalloprotease A69 has promising potential in relation to the preparation of bioactive peptides from peanut protein.

## 1. Introduction

Bioactive peptides, usually containing 2–40 amino acid residues, are peptide compounds that are beneficial to the life activities of organisms or have physiological effects [1,2]. A large number of studies have shown that some oligopeptides have immunoregulatory [3], antibacterial [4], antioxidant [5], antihypertensive [6], and other properties. Bioactive peptides, which are not active in the precursor protein, are usually released from proteins by acid hydrolysis, alkali hydrolysis, or enzymatic hydrolysis, of which enzymatic hydrolysis is the least destructive and the most commonly used. At present, enzymes commonly used for bioactive peptides preparation are pepsin, trypsin, papain, chymotrypsin, Alcalase, and others, all of which are proteases from terrestrial animals or microorganisms. In contrast, few marine proteases have been used in bioactive peptide preparation. Since a lot of marine proteases have been identified, it has important significance to study the potential of marine proteases in the preparation of bioactive peptides.

Protease A69 is a MEROPS M4 family metalloprotease derived from the marine bacterium *Anoxybacillus caldiproteolyticus* 1A02591 [7]. Protease A69 has been found in *Escherichia coli* and *Bacillus subtilis*, and the recombinant protease A69 has been used to prepare bovine bone collagen peptides [7] and soy protein peptides [8]. The prepared collagen peptides have moisturizing and antioxidant properties, and the prepared soybean peptides have angiotensin-converting enzyme (ACE)-inhibitory properties. These studies indicate that protease A69 has good potential in the hydrolyzation of proteins to generate bioactive peptides. Because different proteins have different amino acid sequences, and peptides with different sequences can be released from different proteins by a protease, it is thus necessary to study the potential of protease A69 in the preparation of bioactive peptides from other plant/animal proteins.

In China, peanuts are widely used in the production of cooking oil, and the byproduct of peanut oil extraction is conventionally used in animal feed [9,10]. The byproduct contains 47% to 55% protein and is, therefore, a good source for peanut protein preparation. It has been shown that peanut peptides obtained from peanut protein hydrolysis by terrestrial proteases such as Alcalase and trypsin have antioxidant [11,12], antihypertensive [13,14], and hypoglycemic properties [15], indicating that peanut protein is a good material for bioactive peptide preparation.

This study aimed to evaluate the potential of protease A69 from recombinant *Bacillus subtilis* in the preparation of peanut peptides (PPs) with antioxidant and ACE inhibitory properties. On the basis of optimizing the hydrolysis parameters, a process to prepare peanut peptides (PPs) from peanut protein hydrolysis with A69 was established. The characteristics of and bioactivities associated with the prepared PPs were further analyzed. The results indicate that the marine bacterial metalloprotease A69 has promising application prospects in the preparation of PPs with good nutritional value and high antioxidant and ACE-inhibitory properties.

## 2. Results and Discussion

### 2.1. Optimization of the Hydrolysis Parameters of Protease A69 on Peanut Protein

**Figure 1:**
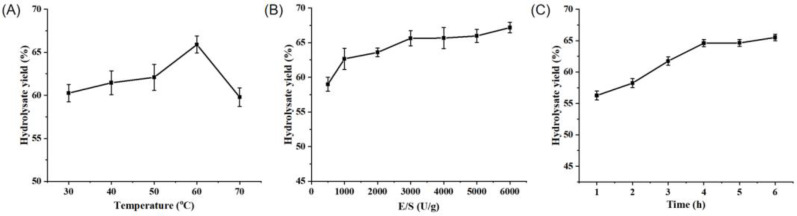
Optimization of the parameters for protease A69 to hydrolyze peanut protein. (**A**) The effect of temperature on the hydrolysate yield. Peanut protein was hydrolyzed by A69 (3000 U g^−1^) for 4 h at pH 7.0 and under different temperatures. (**B**) The effect of the E/S ratio on the hydrolysate yield. Peanut protein was hydrolyzed at 60 °C and pH 7.0 for 4 h by A69 under different E/S ratios. (**C**) The effect of hydrolysis time on the hydrolysate yield. Peanut protein was hydrolyzed by A69 (3000 U g^−1^) at 60 °C and pH 7.0 for different time durations. The graphs show data for triplicate experiments (mean ± SD).

Protease A69 has been previously shown to be associated with the highest activity at 60 °C and pH 7.0 [7]. To investigate the potential of A69 in the hydrolysis of peanut protein and the preparation of PPs, we determined the optimal conditions for the hydrolysis of peanut protein by A69 to produce PPs through the optimization of three hydrolysis parameters, i.e., hydrolysis temperature, enzyme/substrate (E/S) ratio, and hydrolysis time, by single-factor experiments. According to the hydrolysate yield and the proportion of small peptides (<1000 Da) in the produced PPs, the optimal temperature for the hydrolysis of peanut protein by A69 was determined to be 60 °C (Figure 1A and Table 1). At 60 °C and a pH of 7.0, in the E/S range of 500–6000 U g^−1^, the hydrolysate yield and the proportion of small peptides (<1000 Da) in the produced PPs increased continuously and they virtually stabilized after 3000 U g^−1^ (Figure 1B and Table 2). Thus, the optimal E/S ratio was determined to be 3000 U g^−1^. At 60 °C and a pH of 7.0, with 3000 U g^−1^ A69, the hydrolysate yield and the proportion of small peptides (<1000 Da) in the produced PPs increased continuously with time, and they virtually stabilized after 4 h of hydrolysis (Figure 1C and Table 3). Thus, 4 h was regarded as the optimal hydrolysis duration.

Protease A69 has been used to prepare collagen peptides from bovine bone collagen and soy protein peptides from soy protein. The optimal hydrolytic parameters for recombinant A69 from *E. coli* to prepare collagen peptides have been found to be 60 °C, 25 U (collagenolytic activity) g^−1^, and 2 h [7]. The optimal hydrolytic parameters for recombinant A69 from *B. subtilis* to prepare soy protein peptides have been shown to be 60 °C, 4000 U (caseinolytic activity) g^−1^, and 3 h [8]. In this study, the optimal hydrolytic parameters for recombinant A69 from *B. subtilis* to prepare PPs from peanut protein were determined to be 60 °C, 3000 U (caseinolytic activity) g^−1^, and 4 h. Thus, some of the hydrolytic parameters for A69 to prepare bioactive peptides from different proteins are different, maybe due to the differences in protein sequences and structures.

### 2.2. Preparation and Characterization of PPs

**Figure 2:**
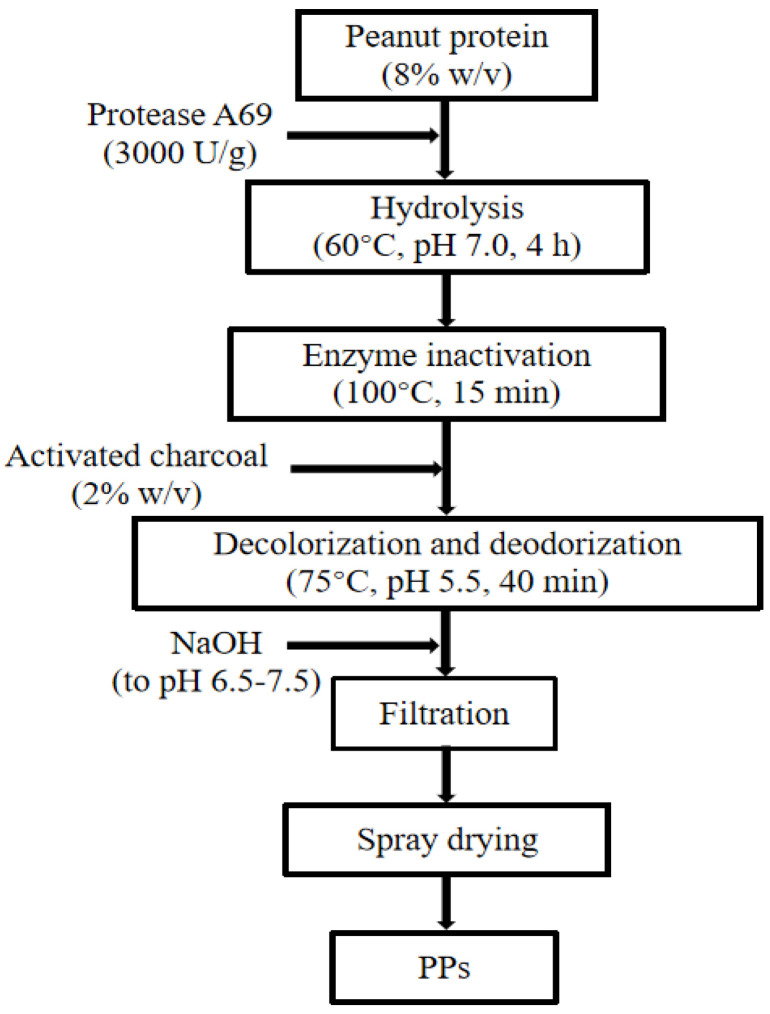
A flow chart of the preparation of PPs with protease A69.

**Figure 3:**
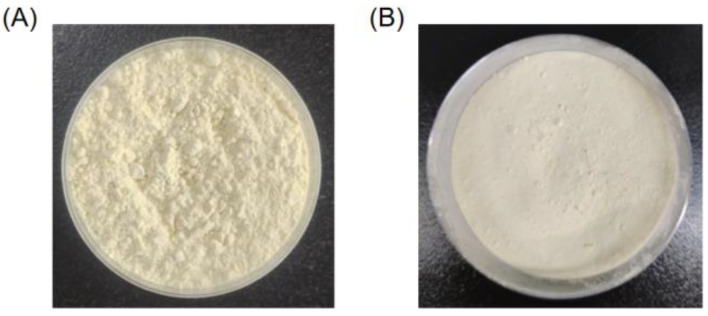
Peanut protein powder and PPs powder. (**A**) Peanut protein powder before enzymatic hydrolysis. (**B**) PPs powder prepared using the process shown in Figure 2.

**Figure 4:**
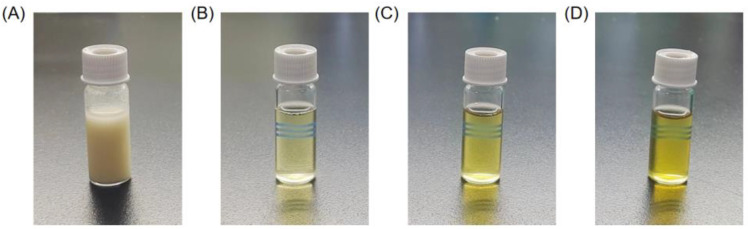
Solubility of peanut protein powder and PPs powder. (**A**) Peanut protein solution, 10% (*w*/*v*). (**B**) PPs solution, 10% (*w*/*v*). (**C**) PPs solution, 20% (*w*/*v*). (**D**) PPs solution, 30% (*w*/*v*).

**Figure 5:**
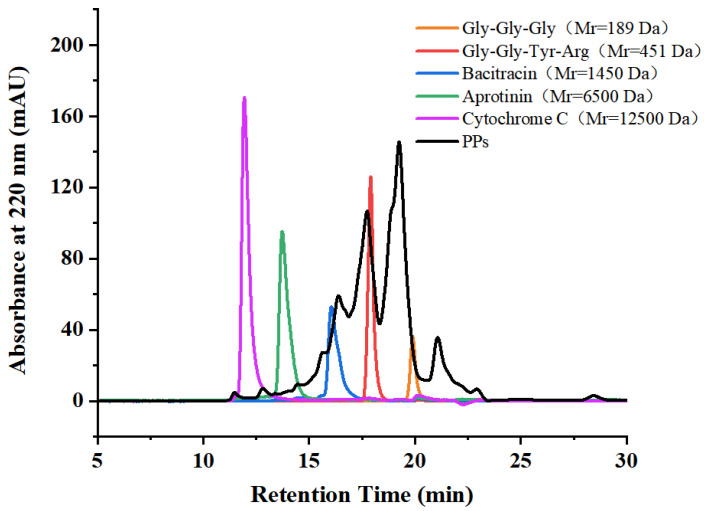
Molecular weight distribution of the prepared PPs analyzed by HPLC.

Based on the optimized enzymatic hydrolysis conditions, we established a process for the preparation of PPs using protease A69 (Figure 2). In this process, the hydrolysis temperature, E/S ratio, and hydrolysis time were 60 °C, 3000 U g^−1^, and 4 h, respectively. Using this process, the hydrolysis efficiency of the peanut protein sample was 64.90% based on the weight of the sample before and after hydrolysis. According to the determined protein contents in the sample before and after hydrolysis, the hydrolysis efficiency of the protein in the peanut protein sample was 86.15 ± 0.42%. Compared to the light yellow, coarse peanut protein powder (Figure 3A), the prepared PPs were much finer and exhibited a milky white color (Figure 3B). While the peanut protein powder was poorly soluble in water (Figure 4A), the PPs at 10–30% (*w*/*v*) content were completely dissolved in water, resulting in clear solutions (Figure 4B–D). Peptides with a molecular weight lower than 3000 Da, 1000 Da, and 500 Da in the PPs accounted for 96.56%, 82.75%, and 54.19%, respectively (Figure 5 and Table 4), indicating that the majority of the PPs were peptides composed of less than 20 amino acid residues. As bioactive peptides usually contain 2–20 amino acid residues [3,4], this result indicates that the prepared PPs may contain a variety of bioactive peptides and may exhibit good biological activity.

### 2.3. Amino Acid Composition of the Prepared PPs

The composition of the prepared PPs with respect to free and total amino acids was analyzed by an automatic amino acid analyzer, and the results are shown in Table 5. Due to the use of hydrochloric acid in the hydrolysis process, Trp was destroyed and not detected. The content of free amino acids in the prepared PPs was less than 3%. Among the total amino acids in the PPs, the most abundant amino acid was Glu, which accounted for 14.71 ± 0.62% of the total. The contents of Pro, Asp, and Arg were also high, accounting for 12.37 ± 0.90%, 7.49 ± 0.69%, and 5.11 ± 0.46%, respectively. It is worth noting that there were seven essential amino acids detected in the PPs, accounting for 18.86 ± 1.37% in total. These data show that the prepared PPs have a good nutritional value.

It has been reported that aromatic amino acid residues in peptides are very important for their ACE-inhibitory properties [16]. As shown in Table 5, the proportion of aromatic amino acid residues, such as Phe and Tyr, in the PPs was relatively high, implying that the PPs may contain ACE-inhibitory peptides. In addition, it has been reported that some hydrophobic amino acid residues, as well as acidic and basic amino acids, are able to act as antioxidants by acting as proton or electron donors [17,18,19]. The PPs contained large amounts of hydrophobic amino acids such as Pro, Phe, Val, Leu, and Ala, as well as acidic amino acids (Glu and Asp) and basic amino acids (Arg, Lys and His) (Table 5), suggesting that the PPs may contain antioxidant peptides.

### 2.4. The Antioxidant Activity of the Prepared PPs

Antioxidants play an important role in human health because they protect the body from damage caused by free radicals [20]. Peptides from food sources are potential natural antioxidants that do not cause significant adverse effects. Peptides exhibiting good radical scavenging usually have antioxidant properties and can be widely used in cosmetics and food fields. At present, a variety of peptides with antioxidant properties have been reported, such as carp peptides [21], rice endosperm peptides [22], croceine croaker peptides [23], oyster peptides [24], and salmon peptides [25].

**Figure 6:**
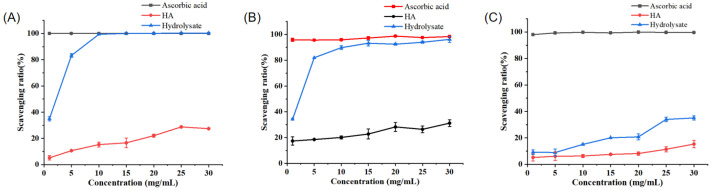
Antioxidant activity of the prepared PPs. (**A**) •OH scavenging capacity of PPs, ascorbic acid, and HA. (**B**) DPPH• scavenging capacity of PPs, ascorbic acid, and HA. (**C**) O_2_^−^• scavenging capacity of PPs, ascorbic acid, and HA.

We analyzed the ability of the prepared PPs to scavenge the radicals DPPH•, •OH, and O_2_^−^•. Among them, the PPs demonstrated the best scavenging ability with respect to •OH. When the concentration of the PPs was equal to or greater than 10 mg mL^−1^, the scavenging activity of the PPs relative to •OH was nearly 100%, similar to that of ascorbic acid (Vc) and much higher than that of hyaluronic acid (HA) (Figure 6A). The PPs also demonstrated high DPPH• scavenging activity, which was close to 90% when the concentration of the PPs was 10 mg mL^−1^ (Figure 6B). The IC_50_ values of the PPs for •OH and DPPH• were 1.50 ± 0.06 and 1.66 ± 0.06 mg mL^−1^, respectively. In contrast, the O_2_^−^• scavenging activity by the PPs was low, attaining only 30% when the concentration of the PPs was 25 mg mL^−1^. These results show that the PPs prepared with A69 demonstrate radical scavenging ability, especially high activity against DPPH• and •OH, suggesting that the PPs contain antioxidant peptides.

Several studies have shown that peanut peptides have radical scavenging properties. At the concentration of 10 mg mL^−1^, the scavenging activity of peanut peptides obtained from papain by Tang et al. was 65.33% with respect to DPPH• and 54.34% with respect to •OH [11]. The peanut protein hydrolysates prepared using pepsin by Ma et al. exhibited high scavenging activity with respect to DPPH• (84.72 ± 0.18%) and •OH (85.62 ± 0.70%) at 50 mg mL^−1^ [12]. Comparatively, the PPs prepared with the marine bacterial protease A69 demonstrated significantly higher DPPH• and •OH scavenging activity than those prepared using papain or pepsin.

It is common for a protein hydrolysate to demonstrate different degrees of scavenging relative to different radicals. In this study, the PPs demonstrated high DPPH• and •OH scavenging activity, but quite low O_2_^−^• scavenging activity. The underlying mechanism for this phenomenon warrants further study.

### 2.5. The ACE-Inhibitory Activity of the Prepared PPs

Angiotensin I-converting enzyme (ACE, EC 3.4.15.1) plays a role in regulating human blood pressure in the renin–angiotensin system, which catalyzes the conversion of inactive prohormone angiotensin I into active hypertension hormone angiotensin II, and plays a role in the degradation of bradykinin, a vasodilator [26,27,28]. Due to the important role of ACE in regulating blood pressure, synthetic inhibitors of this enzyme have been used to treat high blood pressure [29]. However, these inhibitors usually have some side effects. Therefore, it is important to find safer natural sources of ACE inhibitors. At present, a variety of peptides with ACE-inhibitory properties have been found, such as soybean peptides [30], spinach peptides [31], wheat gliadin peptides [32], bitter melon seed peptides [33], and lizard fish peptides [34].

**Figure 7:**
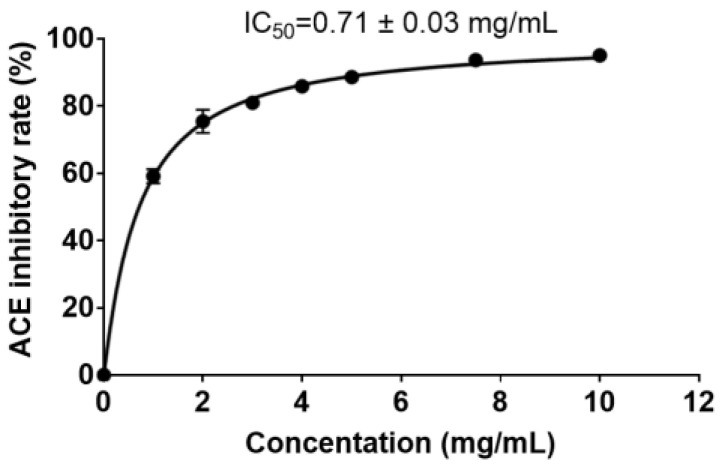
The ACE-inhibitory rate of PPs of different concentrations.

In order to determine the ACE-inhibitory activity of the PPs prepared with protease A69, the ACE-inhibitory rates of PPs of different concentrations were measured (Figure 7), and the IC_50_ value was determined to be 0.71 ± 0.03 mg mL^−1^. Thus, the PPs demonstrated remarkable ACE-inhibitory activity and likely contained ACE-inhibitory peptides.

Several peanut hydrolysates have been reported to have ACE-inhibitory properties. Zhang et al. used a combination of enzymes to hydrolyze peanut protein, and the hydrolysate showed high ACE-inhibitory activity, with an IC_50_ value of 0.548 mg mL^−1^ [13]. The peanut hydrolysate prepared with Alcalase by Guang et al. exhibited a high ACE-inhibitory rate, with an IC_50_ value of 0.134 mg mL^−1^ [14]. Comparatively, the ACE-inhibitory activity of the PPs prepared with the marine bacterial protease A69 was lower than those reported above.

### 2.6. Identification of Antioxidant Peptides and ACE-Inhibitory Peptides from the Prepared PPs

The peptide sequences in the prepared PPs were determined by LC-MS/MS, and a total of 1950 peptide sequences from peanut proteins were detected. Based on the determined peptide sequences, the residue frequencies of P1 and P1’ sites of the peptide bonds hydrolyzed by A69 were calculated (Table 6). It has been reported that enzymes in the M4 family preferentially hydrolyze peptide bonds with hydrophobic amino acid residues, and that the P1’ site is most likely to be an aromatic amino acid residue [35,36]. As shown in Table 6, the protease A69 preferentially hydrolyzed peptide bonds in peanut proteins with hydrophobic residues at the P1’ site, such as Leu, Val, Ile, Ala, and Phe, similar to other enzymes in the M4 family. It has been reported that peptides whose P1’ position is hydrophobic (Leu, Val, Ile, Gly, Ala, Phe, and Pro) [37], aromatic (Phe, Tyr, and Trp), Pro or aliphatic (Leu, Ile, Ala, and Met) [38] tend to have ACE-inhibitory properties. Cheung et al. reported that peptides with high ACE-inhibitory activity contained Pro, Phe, or Tyr at the C-terminal and Val or Ile at the N-terminal [39]. Regarding the correlation between the structure and activity of antioxidant peptides, Li et al. reported that polar/charged amino acids such as Arg or His at the P1 position contribute to the antioxidant activity [40]. It has also been reported that one or two residues of His, Pro, Met, Cys, Tyr, Trp, and Phe could enhance the activity of antioxidant peptides [41].

A variety of ACE-inhibitory peptide sequences and antioxidant peptide sequences have been reported, most of which are deposited in the AHTPDB database and the AODB database, respectively. By searching the published ACE-inhibitory peptides in the AHTPDB database, 19 ACE-inhibitory peptides from different sources were identified in our prepared PPs, including four dipeptides, 14 tripeptides, and one tetrapeptides (Table 7). By searching the published antioxidant peptides in the AODB database, 15 antioxidant peptides from different sources were identified in our prepared PPs, including one dipeptides, 12 tripeptides, and two tetrapeptides (Table 8). These data indicate that marine bacterial protease A69 can induce the release of a variety of ACE-inhibitory peptides and antioxidant peptides from peanut proteins; it can be concluded, therefore, that the prepared PPs demonstrate ACE-inhibitory activity and antioxidant activity. As shown in Table 7 and Table 8, these ACE-inhibitory peptides and antioxidant peptides are derived from a variety of plant and animal sources but not peanut. In addition, the IC_50_ values of these ACE-inhibitory peptides show remarkable differences, and so does the antioxidant activity exhibited by the antioxidant peptides. Notably, the activities of these peptides have mainly been detected in cell-free models; therefore, more in vitro or in vivo experiments would be necessary to confirm the potential effects of these peptides.

So far, five ACE-inhibitory peptides have been identified in peanuts, including KAFR [14], IKP [42], IEY [43], KLYMRP [44], and CVTPALR [45], as well as five antioxidant peptides, including PGCPST [12], TPA [46], SP [46], I/LPS [46], and YGS [47]. However, the sequences of the antioxidant peptides and ACE-inhibitory peptides identified from the PPs in this study (Table 7 and Table 8) are all different from those reported in peanuts, indicating that the 19 ACE-inhibitory peptides (Table 7) and the 15 antioxidant peptides (Table 8) are the first to be identified in peanuts.

**Table 7:** The ACE-inhibitory peptides identified in the prepared PPs.

Sequence	Molecular Weight (Da)	Source	IC_50_ (μmol L^−1^)	References
IG	188.23	Milk	<20	[16]
IG	188.23	Cereals storage protein	-	[48]
IG	188.23	Pork sarcoplasmic proteins	-	[49]
GA	146.15	-	<20	[16]
GA	146.15	Pork sarcoplasmic proteins	-	[49]
GA	146.15	Cereals storage protein	-	[48]
GA	146.15	-	2000	[39]
KW	332.4	Milk	-	[50]
KW	332.4	-	<20,000	[16]
KW	332.4	Wakame (*Undaria pinnatifida*)	2.7–43.7	[51]
KW	332.4	Pork sarcoplasmic proteins	-	[49]
KW	332.4	Cereals storage protein	-	[48]
KW	332.4	Fish (Sardine (*Sardina pilchardus* muscle))	1.63	[52]
KW	332.4	Wakame (*Undaria pinnatifida*)	10.8	[53]
KW	332.4	Synthesized	7.8	[54]
PG	172.18	Pork sarcoplasmic proteins	-	[49]
PG	172.18	Cereals storage protein	-	[48]
PG	172.18	-	17,000	[39]
PG	172.18	-	<20	[16]
PLG	285.34	Fish (Alaska pollack (*Theragra chalcogramma*))	4.74	[55]
PLG	285.34	Cereals storage protein	-	[48]
VLP	327.42	Milk	-	[50]
VLP	327.42	Pork sarcoplasmic proteins	-	[49]
VLP	327.42	Cereals (Finnish)	0.46	[48]
VLP	327.42	-	<20	[16]
VFPS	448.52	Synthesized	0.46	[54]
HIR	424.5	Milk derived	953	[16]
PPL	325.41	Insect	>1000	[56]
MKP	374.5	Milk-derived	<10	-
VRW	459.55	Milk-derived	<10	-
TVY	381.43	Milk (digested milk products)	15	[16]
GAW	332.36	Milk-derived	-	[16]
QQI	387.44	Milk–cheese (goat milk protein and cheeses)	-	[57]
IPQ	356	Milk (sodium caseinate)	-	[58]
YGG	295.3	-	<20	[16]
GYK	366.42	Bovine beta lactoglobulin	160	-
YPR	434.5	Sake and sake lees	16.5	[59]
VIF	377.48	Fish (sea bream scale)	7.5	[60]

**Table 8:** The antioxidant peptides identified in the prepared PPs.

Sequence	Average Molecular Weight (Da)	Source	Antioxidant Activity	References
LLPH	478.58	Designed peptide	-	[61]
RHI	424.5	Synthesis peptide	The trolox equivalent antioxidant capability (TEAC) of the peptide was 0.189 (mMTE)	[62]
RWI	473.57	Synthesis peptide	The trolox equivalent antioxidant capability (TEAC) of the peptide was 0.702 (mMTE)	[62]
YVL	393.48	Bovine milk protein	The peptide showed the ACE-inhibitory activity (IC_50_ > 1000 μmol L^−1^) and oxygen radical absorption capacity (0.96 ± 0.04 μmol trolox equivalent per μmol of peptide) of chemically synthesized peptides	-
TY	282.29	Potato protein	-	[63]
YGG	295.29	Bovine whey protein	The peptide YGG showed very low ABTS+ free radical scavenging activity (75%) at the concentration of 100 μM	[64]
FVPH	498.57	Chickpea legumin	-	[65]
PEQ	372.37	bovine whey protein (b-lactoglobulin)	The peptide showed antioxidant activity of 0.22 ± 0.02 mmol Fe^3+^ mol^−1^ (FARP)	[66]
HIR	424.5	Bovine whey protein (b-lactoglobulin)	The peptide showed antioxidant activity of 1.31 ± 0.12 mmol Fe^3+^ mol^−1^ (FARP)	[66]
TCG	279.31	Bovine whey protein (b-lactoglobulin)	The peptide showed antioxidant activity of 4.67 ± 0.17 mmol Fe^3+^ mol^−1^ (FARP)	[66]
KPT	344.4	Bovine whey protein (b-lactoglobulin)	The peptide showed antioxidant activity of 0.52 ± 0.06 mmol Fe^3+^ mol^−1^ (FARP)	[66]
PEG	301.29	Bovine whey protein (b-lactoglobulin)	The peptide showed antioxidant activity of 0.19 ± 0.04 mmol Fe^3+^ mol^−1^ (FARP)	[66]
NGE	318.28	Bovine whey protein (b-lactoglobulin)	The peptide showed antioxidant activity of 0.50 ± 0.09 mmol Fe^3+^ mol^−1^ (FARP)	[66]
PAV	285.34	Bovine whey protein (b-lactoglobulin)	The peptide showed antioxidant activity of 1.10 ± 0.02 mmol Fe^3+^ mol^−1^ (FARP)	[66]
AVF	335.4	Bovine whey protein (b-lactoglobulin)	The peptide showed antioxidant activity of 2.28 ± 0.06 mmol Fe^3+^ mol^−1^ (FARP)	[66]

## 3. Materials and Methods

### 3.1. Experimental Materials

The peanut protein powder was kindly provided by Dezhou Lanli Biotechnology Limited Company (Dezhou, China). The protease A69 was produced using recombinant *Bacillus subtilis*, as previously reported [8]. Aprotinin, cytochrome C, salicylic acid, pyrogallol, ACE, hippuric acid, and Hip-His-Leu (HHL) were purchased from Sigma (St Louis, MO, USA). Bacitracin and H_2_O_2_ were purchased from Aladdin (Shanghai, China). Tetrapeptide GGYR and tripeptide GGG were synthesized by Qiangyao Co., Ltd. (Shanghai, China). Ascorbic acid was purchased from Sinopharm Chemical Reagent Co., Ltd. (Shanghai, China). HA was purchased from Shandong Freda Bioeng Co., Ltd. (Jinan, China). DPPH• was purchased from Tokyo Chemical Industry (Tokyo, Japan). Other chemicals were of analytical grade and commercially available.

### 3.2. Optimization of the Hydrolytic Parameters of Protease A69 with Respect to Peanut Protein

The activity of A69 was determined using casein as the substrate by following the method previously reported [67]. One unit (1 U) was defined as the amount of enzyme that released 1 µg of tyrosine from casein per min.

The hydrolysis parameters of peanut protein produced by A69, including hydrolysis temperature, hydrolysis time, and enzyme/substrate (E/S) ratio, were optimized according to the methods previously used in bovine bone collagen hydrolysis by A69 [7] with some modifications. To determine the optimal hydrolysis temperature, 1 g of peanut protein powder in 25 mL ddH_2_O was hydrolyzed with A69 (3000 U g^−1^) at different temperatures (30 °C, 40 °C, 50 °C, 60 °C, or 70 °C) for 4 h and the solution was constantly stirred (180 rpm). To determine the optimal hydrolysis time, 1 g of peanut protein powder in 25 mL ddH_2_O was hydrolyzed with A69 at an E/S ratio of 3000 U g^−1^ at 60 °C and the solution was constantly stirred (180 rpm) for different time durations (1–6 h). To determine the optimal E/S ratio, 1 g of peanut protein powder in 25 mL ddH_2_O was hydrolyzed at 60 °C and the solution was constantly stirred (180 rpm) for 4 h with A69 under different E/S ratios (500 U g^−1^, 1000 U g^−1^, 2000 U g^−1^, 3000 U g^−1^, 4000 U g^−1^, 5000 U g^−1^, or 6000 U g^−1^). Because the optimal pH for A69 activity is 7.0 [7], all the hydrolytic reactions were performed at pH 7.0. After hydrolysis, the mixture was incubated at 100 °C for 15 min to inactivate the enzyme, and then centrifuged at 4 °C and 10,000× *g* for 30 min. The supernatant containing the PPs and the residual peanut protein were separately freeze-dried. The freeze-dried residual peanut protein was weighed, and the weight was used to calculate the hydrolysate yield using the formula below. The obtained PPs were dissolved in ddH_2_O to prepare 5 mg mL^−1^ of sample solution for peptide molecular weight distribution analysis by HPLC (Shimadzu, Kyoto, Japan) on a TSK gel G2000 SWXL column (7.8 × 300 mm; Tosoh, Tokyo, Japan). The column was eluted with 45% acetonitrile containing 0.1% (*v*/*v*) trifluoroacetic acid at a flow rate of 0.5 mL min^−1^. Peptide signals were monitored at 220 nm. The molecular weight standard samples used included cytochrome C (12,500 Da), aprotinin (6500 Da), bacitracin (1450 Da), tetrapeptide GGYR (451 Da), and tripeptide GGG (189 Da). The proportion of peptides with different molecular weights (<500 Da, <1000 Da, 1000–3000 Da, 3000–5000 Da, 5000–10,000 Da, and >10,000 Da) in the PPs was determined as the percentage of the area of the corresponding molecular weight range to the total chromatograph area. The hydrolysate yield of peanut protein was calculated using the formula:

Hydrolysate yield (%) = (*W*_a_ − *W*_b_)/*W*_a_ × 100, (1)

where *W*_a_ and *W*_b_ are the dry weights of the peanut protein samples before and after being hydrolyzed, respectively.

### 3.3. Preparation of PPs Using A69

To prepare PPs, 80 g of peanut protein in 1 L ddH_2_O was hydrolyzed with A69 (3000 U g^−1^) at 60 °C, pH 7.0, and the solution was constantly stirred (180 rpm) for 4 h. During hydrolysis, the pH of the mixture was maintained at 7.0 with 10 M NaOH solution. After hydrolysis, the reaction was terminated by incubating the mixture at 100 °C for 15 min to inactivate the protease. Then, the temperature and pH of the mixture were adjusted to 75 °C and 5.5, respectively, and 2% activated carbon (*w*/*v*) was added to the mixture. The mixture was incubated at 75 °C and constantly stirred (180 rpm) for 40 min to remove color and odor. After that, the pH of the mixture was adjusted to 6.5–7.0 using 16 M phosphoric acid, and then the mixture was centrifuged and filtered with a 0.22 µm filter membrane. The filtrate was collected and spray dried. The resulting powder constituted the PPs prepared with protease A69.

### 3.4. Characterization of the Prepared PPs

The PPs powder was dissolved in ddH_2_O to prepare 5 mg mL^−1^ of sample solution. The peptide molecular weight distribution of the PPs was analyzed by HPLC as described above.

To analyze the water solubility of the PPs powder, 10%, 20%, and 30% (*w*/*v*) of PPs solutions were prepared by dissolving the PPs powder in ddH_2_O. A total of 10% (*w*/*v*) of peanut protein solution was used as the control.

The composition of free amino acids and total amino acids in the PPs was analyzed with the method described previously [7]. Briefly, 5 mg of the prepared PPs powder was dissolved in 1 mL ddH_2_O containing trifluoroacetic acid (1%, *v*/*v*) and the solution was divided into two samples for the analysis of free amino acids and total amino acids. For free amino acid analysis, sulfosalicylic acid (4%, *w*/*v*) was added to the sample, and the sample was incubated at 25 °C for 30 min, followed by centrifugation (10,000× g) at 4 °C for 10 min. The supernatant was collected and used for free amino acid analysis. For total amino acid analysis, the sample was hydrolyzed at 110 °C using 6.0 M HCl for 22 h. Subsequently, the HCl was evaporated from the sample by rotation. The sample was redissolved in ddH_2_O and used for total amino acid analysis. An automatic amino acid analyzer, HITACHI 835 (HITACHI, Tokyo, Japan), was used to analyze the amino acid composition of the two samples.

### 3.5. Determination of Protein Content

The protein content of the peanut protein samples before and after A69 hydrolysis was determined using the colorimetric method according to the National Standard of the People’s Republic of China for determination of protein in foods (GB 5009.168-2016) [68]. Briefly, the sample (0.1 g) was mixed with 0.1 g of CuSO_4_, 1 g of K_2_SO_4_, and 5 mL (*V*_1_) of 18.4 M H_2_SO_4_ and the mixture was heated on a light wave furnace. After assuming a clear blue-green coloration, the mixture solution was further heated for 0.5 h. After cooling, the volume of the solution was fixed to 100 mL (*V*_2_) with ddH_2_O. The sample containing only CuSO_4_, K_2_SO_4_, and H_2_SO_4_ was used as the blank control. Next, 3 mL (*V*_3_) of the sample (containing two drops of 1 g L^−1^ p-nitrophenol indicator solution) was neutralized by 10 M NaOH until the solution became yellow. The solution was then rendered colorless with 1 M acetic acid and diluted to 5 mL (*V*_4_) with ddH_2_O. A total of 200 µL of the sample solution was heated with 400 µL of sodium-acetate–acetic-acid buffer solution (pH 4.8) and 400 µL of formaldehyde–acetone solution in a 100 °C water bath for 15 min. After cooling to room temperature, the absorbance of the solution was measured at 400 nm. The standard curve was made with ammonia nitrogen standard solutions of different concentrations (0 µg mL^−1^, 5 µg mL^−1^, 10 µg mL^−1^, 20 µg mL^−1^, 40 µg mL^−1^, 60 µg mL^−1^, 80 µg mL^−1^, 100 µg mL^−1^). The protein content of the sample was calculated as follows:

(2) $$X=\frac{c-c_{0}}{m\times \frac{V_{1}}{V_{2}}\times \frac{V_{3}}{V_{4}}\times 1000\times 1000}\times 100\times F$$

where *X* is the protein content of the sample (g 100 g^−1^), *c* is the nitrogen content of the sample solution (μg), *c*_0_ is the nitrogen content of the blank solution (μg), *V*_1_, *V*_2_, *V*_3_, and *V*_4_ as shown above, *m* is the sample quantity (g), and *F* is the conversion coefficient of nitrogen to protein which was taken as 5.46 for peanut and its products.

### 3.6. Assay of the Antioxidant Activity of the Prepared PPs

The antioxidant activity of the prepared PP samples was analyzed by measuring its free radical scavenging activity toward DPPH•, •OH, and O_2_^−^• according to the methods described by Cheng et al. [7]. Vc and HA were used as two positive controls. To determine the DPPH• scavenging activity, 100 µL of PPs (1 mg mL^−1^, 5 mg mL^−1^, 10 mg mL^−1^, 15 mg mL^−1^, 20 mg mL^−1^, 25 mg mL^−1^, or 30 mg mL^−1^) was reacted with 200 µL of 100 µM DPPH• (dissolved in 50% ethanol solution) at 25 °C in the dark for 40 min. Then, the absorbance of the reaction solution was measured at 525 nm. Under the same reaction conditions, the DPPH• solution replaced by an equal volume of 50% ethanol solution was used as the background group, and the sample replaced by an equal volume of ddH_2_O was used as the blank control group. To determine the •OH scavenging activity, 200 µL of PPs (1 mg mL^−1^, 5 mg mL^−1^, 10 mg mL^−1^, 15 mg mL^−1^, 20 mg mL^−1^, 25 mg mL^−1^, or 30 mg mL^−1^) was mixed with 200 µL of 9 mM FeSO_4_ (dissolved in ddH_2_O) and 200 µL of 9 mM salicylic acid (dissolved in anhydrous ethanol) at 37 °C. Then, 200 µL of 8.8 mM H_2_O_2_ solution was added to the mixture, and the reaction was conducted at 37 °C, in the darkness, for 30 min. The absorbance of the reaction solution was measured at 510 nm. Under the same reaction conditions, 8.8 mM H_2_O_2_ replaced by an equal volume of ddH_2_O was used as the background group, and the sample replaced by an equal volume of ddH_2_O was used as the blank control group. To determine the O_2_^−^• scavenging activity, 200 µL of PPs (1 mg mL^−1^, 5 mg mL^−1^, 10 mg mL^−1^, 15 mg mL^−1^, 20 mg mL^−1^, 25 mg mL^−1^, or 30 mg mL^−1^) was mixed with 900 µL of 50 mM Tris-HCl buffer (pH 8.2) and 80 µL of 25 mM pyrogallol solution (dissolved in 10 mM HCI). The reaction was conducted at 25 °C, in the darkness, for 5 min. The reaction was terminated by adding 200 µL of 8 mM HCl. The absorbance of the reaction solution was measured at 320 nm. Under the same reaction conditions, 80 µL of 10 mM HCl instead of the pyrocatechol solution was used as the background group, and an equal volume of ddH_2_O instead of the sample was used as the blank control group. The free radical scavenging activity (D) was calculated by using the following formula:

*D* (%) = [1 − (*A_i_* − *A_j_*)/*A*_0_] × 100, (3)

where *A_i_* is the absorbance of the sample, *A_j_* is the background absorbance of the sample, and *A*_0_ is the absorbance of the blank control.

### 3.7. Assay of the ACE-Inhibitory Activity of the Prepared PPs

The ACE-inhibitory activity of the prepared PPs was analyzed using the method described by Wang et al. [69]. The PPs powder was dissolved in 0.1 M boric acid buffer solution containing 0.3 M NaCl (pH 8.3) to prepare PPs solutions of different concentrations (1 mg mL^−1^, 2 mg mL^−1^, 3 mg mL^−1^, 4 mg mL^−1^, 5 mg mL^−1^, 7.5 mg mL^−1^, and 10 mg mL^−1^). The PPs solutions (25 μL) of different concentrations were mixed with 25 μL of HHL (5 mM). The mixture was incubated at 37 °C for 5 min. After that, 25 μL of 0.1 U mL^−1^ ACE was added to the mixture, which was further incubated at 37 °C for 30 min. Then, 25 μL of trifluoroacetic acid solution (4%) was added to the mixture to stop the reaction. Boric acid buffer instead of the PPs solution in the mixture served as the blank control. The amount of hippuric acid released from HHL hydrolysis by ACE was determined by HPLC (Shimadzu, Kyoto, Japan) on a SunFire C18 column (4.6 × 250 mm, 5 μm; Waters, Milford, MA, USA), which was monitored at 214 nm. The column was eluted with a mobile phase of acetonitrile:H_2_O:trifluoroacetic acid = 40:60:0.1 at a flow rate of 1 mL min^−1^. The ACE-inhibitory rate (R) was calculated using the following formula:

*R* (%) = (1 − *B*)/*A* × 100, (4)

where *A* and *B* are the peak areas of hippuric acid in blank and experimental samples, respectively. A 100% ACE activity was defined as the amount of hippuric acid released from HHL hydrolysis by ACE in the absence of inhibitors. The IC_50_ of a sample was defined as the amount of sample required to inhibit ACE activity by 50%.

### 3.8. LC-MS/MS Analysis of the Prepared PPs

The prepared PPs were analyzed by mass spectrometry using Q Exactive™ Hybrid Quadrupole-Orbitrap™ Mass Spectrometer (Thermo Fisher Scientific, Waltham, MA, USA) and a sequence database search was performed in Beijing biotech-pack Scientific Co., Ltd. (Beijing, China). A total of 1 g of PPs powder was dissolved in 100 μL of 50 mM NH_4_HCO_3_. After being reduced by 1 μL of 10 mM DTT at 56 °C for 1 h and alkylated by 10 μL of 0.5 M IAM at room temperature, in the dark, for 40 min, the peptides were desalted using a self-priming desalting column, and the solvent was evaporated in a vacuum centrifuge at 45 °C. Then, the peptides were dissolved in 100 μL of 0.1% formic acid for LC-MS/MS analysis. The peptides in the sample were separated on LC with an inorganic phase A (0.1% formic acid) and an organic phase B (80% acetonitrile and 0.1% formic acid) by Reprosil-Pur 120 C18-AQ 3 μm (1.9 μm, 100 Å, Dr. Maisch GmbH, Tübingen, BW, Germany) on the Easy-nLC 1200 system (ThermoFisher Scientific, Waltham, MA, USA). The flow rate was 600 nL min^−1^. The LC linear gradient program used was: from 4% to 8% B for 3 min, from 8% to 28% B for 86 min, from 28% to 40% B for 20 min, from 40% to 95% B for 1 min, and from 95% to 95% B for 10 min. Then, mass spectrometry was performed using Q Exactive™ Hybrid Quadrupole-Orbitrap™ Mass Spectrometer. The raw MS files were analyzed and searched against protein databases based on the species in the sample using Byonic. The parameters were set as follows: the protein modifications were carbamidomethylation (C) (fixed), oxidation (M) (variable), and acetyl (N-term) (variable), the enzyme specificity was set to non-specific, the maximum missed cleavages were set to 3, the precursor ion mass tolerance was set to 20 ppm, and MS/MS tolerance was 0.02 Da. Only peptides identified with high confidence were chosen for downstream protein identification analysis.Based on the determined peptide sequences, the residue frequencies of the P1 and P1’ sites of the peptide bonds hydrolyzed by A69 were calculated. The peptide sequences with ACE-inhibitory activity deposited in the AHTPDB database (https://webs.iiitd.edu.in/raghava/ahtpdb/, accessed on 1 June 2024) were searched in the determined peptide sequences of the prepared PPs. The peptide sequences with antioxidant activity deposited in the AODB database (https://aodb.idruglab.cn/, accessed on 1 June 2024) were searched in the determined peptide sequences of the prepared PPs.

## 4. Conclusions

In the present study, a process to prepare PPs using the marine bacterial metalloprotease A69 was established by optimizing the hydrolysis parameters. With this process, the hydrolysis efficiency of peanut protein powder reached 86.15%, and the prepared PPs powder was fine and had good solubility. The PPs contained 17 kinds of amino acids and exhibited a high content of peptides with a molecular weight lower than 1000 Da (>80%) and 3000 Da (>95%). The PPs showed remarkable ACE-inhibitory activity, with an IC_50_ of 0.71 mg mL^−1^, and showed a great scavenging ability with respect to •OH and DPPH•, with IC_50_ values of 1.50 mg mL^−1^ and 1.66 mg mL^−1^, respectively. Moreover, 19 ACE-inhibitory peptides and 15 antioxidant peptides were identified in the PPs. These results indicate that the PPs prepared with A69 have a good nutritional value as well as antioxidant and antihypertensive effects, and may be used as a functional food additive. The findings in this study suggest that the marine bacterial metalloprotease A69 has potential in the preparation of bioactive peptides from peanut protein.

## Figures and Tables

**Table 1:** Molecular weight distribution of the PPs from peanut protein hydrolysis by A69 under different hydrolysis temperatures.

MW Range (Da)	Content (%)				
	30 °C	40 °C	50 °C	60 °C	70 °C
>10,000	2.84	2.80	0.84	0.29	1.24
5000–10,000	6.79	6.20	1.86	0.69	3.80
3000–5000	3.26	3.05	1.60	1.45	5.38
1000–3000	18.21	17.87	15.11	15.20	22.54
<1000	68.90	70.08	80.59	82.37	67.04
<500	43.76	45.73	52.16	53.35	42.17

**Table 2:** Molecular weight distribution of the PPs from peanut protein hydrolysis by A69 under different E/S ratio.

MW Range (Da)	Content (%)						
	500 U/g	1000 U/g	2000 U/g	3000 U/g	4000 U/g	5000 U/g	6000 U/g
>10,000	26.83	13.66	5.37	0.87	0.30	0.22	0.45
5000–10,000	4.69	5.71	3.54	0.58	1.76	1.98	1.13
3000–5000	3.94	3.22	2.40	1.43	1.70	1.75	1.68
1000–3000	11.05	12.47	13.85	14.80	10.69	12.50	12.91
<1000	53.49	64.94	74.84	82.32	85.55	83.55	83.83
<500	39.12	43.85	47.45	53.97	54.58	54.18	55.13

**Table 3:** Molecular weight distribution of the PPs from peanut protein hydrolysis by A69 for different hydrolysis time durations.

MW Range (Da)	Content (%)					
	1 h	2 h	3 h	4 h	5 h	6 h
>10,000	18.29	6.68	3.01	0.78	0.61	0.41
5000–10,000	3.36	2.19	1.36	0.89	0.85	0.55
3000–5000	2.29	1.99	1.90	1.92	2.08	1.98
1000–3000	8.38	12.59	14.80	14.19	14.49	14.33
<1000	67.68	76.55	78.93	82.22	81.97	82.73
<500	42.18	45.51	48.63	52.26	53.26	53.20

**Table 4:** Proportion of peptides with different molecular weights in the prepared PPs based on HPLC analysis.

MW Range (Da)	Content (%)
>10,000	0.00
5000–10,000	0.89
3000–5000	2.55
1000–3000	13.81
<1000	82.75
<500	54.19

**Table 5:** The amino acid composition and content of the PPs prepared using protease A69 ^a^.

Amino Acids	Free Amino Acids (g/100 g)	Total Amino Acids (g/100 g)
Asp	0.005 ± 0.001	7.491 ± 0.688
Thr	0.023 ± 0.002	2.000 ± 0.181
Ser	0.001 ± 0.001	2.786 ± 0.220
Glu	0.389 ± 0.032	14.705 ± 0.616
Gly	0.019 ± 0.002	2.606 ± 0.254
Ala	0.147 ± 0.013	2.922 ± 0.284
Val	0.167 ± 0.011	3.706 ± 0.257
Cys	0.129± 0.004	2.461 ± 0.036
Met	0.053 ± 0.004	0.781 ± 0.052
Ile	0.032 ± 0.003	1.849 ± 0.166
Leu	0.177 ± 0.016	3.526 ± 0.329
Tyr	0.314 ± 0.020	3.106 ± 0.073
Phe	0.546 ± 0.042	3.942 ± 0.188
Lys	0.113 ± 0.007	3.058 ± 0.193
His	0.066 ± 0.004	1.430 ± 0.090
Arg	0.109 ± 0.010	5.108 ± 0.461
Pro	0.323 ± 0.041	12.373 ± 0.900
Trp ^b^	-	-
Total	2.612 ± 0.209	73.848 ± 4.962

^a^ Human essential amino acids are shown in bold. ^b^ Trp was not detectable because it was destroyed in the process of acid hydrolysis. The data shown in the table are from triplicate experiments (mean ± SD).

**Table 6:** Residue frequency at the P1 and P1’ sites of the peptide bonds in peanut proteins hydrolyzed by A69.

Amino Acid	P1	Content ^$^ (%)	Amino Acid	P1’	Content ^$^ (%)
Leu(L)	554	14.25	Leu(L)	600	15.44
Phe(F)	467	12.01	Val(V)	392	10.09
Ile(I)	264	6.79	Ser(S)	370	9.52
Ala(A)	246	6.33	Thr(T)	362	9.32
Gln(Q)	217	5.58	Ile(I)	354	9.11
Val(V)	213	5.48	Gly(G)	225	5.79
Ser(S)	210	5.40	Ala(A)	218	5.61
Thr(T)	199	5.12	Glu(E)	173	4.45
Lys(K)	195	5.02	Phe(F)	169	4.35
Arg(R)	185	4.76	Arg(R)	166	4.27
Gly(G)	169	4.35	Lys(K)	140	3.60
Glu(E)	166	4.27	Pro(P)	132	3.40
Asn(N)	136	3.50	Gln(Q)	125	3.22
Pro(P)	132	3.40	Asn(N)	123	3.17
Tyr(Y)	131	3.37	Asp(D)	104	2.68
Met(M)	121	3.11	His(H)	63	1.62
Asp(D)	109	2.80	Met(M)	63	1.62
His(H)	102	2.62	Tyr(Y)	53	1.36
Trp(W)	42	1.08	Trp(W)	28	0.72
Cys(C)	30	0.77	Cys(C)	26	0.67
Total	3888	100.00	Total	3886	100.00

**^$^** The proportion of the occurrence number of a residue in position P1 or P1’ relative to that of all residues at position P1 or P1’ of the peptide bonds hydrolyzed by A69 deduced from the 1950 peptides detected by LC-MS/MS.

## Data Availability

The original data presented in the study are included in the article; further inquiries can be directed to the corresponding author.
